# Steroid Hormone Secretion Over the Course of the Perimenopause: Findings From the Swiss Perimenopause Study

**DOI:** 10.3389/fgwh.2021.774308

**Published:** 2021-12-14

**Authors:** Jessica Grub, Hannah Süss, Jasmine Willi, Ulrike Ehlert

**Affiliations:** ^1^Clinical Psychology and Psychotherapy, University of Zurich, Zurich, Switzerland; ^2^University Research Priority Program Dynamics of Healthy Aging, University of Zurich, Zurich, Switzerland

**Keywords:** hormone fluctuations, steroid hormones, perimenopause, sex steroids, menopausal transition

## Abstract

**Background:** Perimenopause is characterized by a decline in the steroid hormones, estradiol, and progesterone. By contrast, the steroid hormone cortisol, a marker of the hypothalamic–pituitary–adrenal (HPA) axis, increases. Recent longitudinal studies reported fluctuations in steroid hormone levels during perimenopause, and even increases in estradiol levels. To understand these confounding results, it is necessary to conduct a longitudinal, highly standardized assessment of steroid hormone secretion patterns in perimenopausal women.

**Methods:** This longitudinal study investigated 127 perimenopausal women aged 40–56 years for 13 months. Estradiol, progesterone, and cortisol were assessed using saliva samples, which were collected for two (during months 2 and 12 for estradiol and progesterone) or three (during months 2, 7, and 12 for cortisol) non-consecutive months over the course of the study. A total of 14 saliva samples per participant were analyzed to investigate the courses of estradiol and progesterone. Cortisol awakening response and fluctuations of cortisol throughout the day were measured using a total of 11 saliva samples per participant (on awakening, +30 min, +60 min, at 12:00 p.m., and before going to bed) for months 2, 7, and 12.

**Results:** Multilevel analyses revealed variance in intercept and slope across participants for estradiol [intercept: *SD* = 5.16 (95% CI: 4.28, 6.21), slope: *SD* = 0.50 (95% CI: 0.39, 0.64)], progesterone [intercept: *SD* = 34.77 (95% CI: 25.55, 47.31), slope: *SD* = 4.17 (95% CI: 2.91, 5.99)], and cortisol (intercept: *SD* = 0.18 (95% CI: 0.14, 0.23), slope: *SD* = 0.02 (95% CI: 0.01, 0.02)]. Time predicted cortisol levels [*b* = −0.02, *t*_(979)_ = −6.63, *p* < 0.0001]. Perimenopausal status (early vs. late) did not predict estradiol [*b* = −0.36, *t*_(1608)_ = −0.84, *p* = 0.400], progesterone [*b* = −4.55, *t*_(1723)_ = −0.87, *p* = 0.385], or cortisol [*b* = 0.01, *t*_(1124)_ = 0.61, *p* = 0.542] scores over time.

**Discussion:** Our results are consistent with previous findings emphasizing highly individual fluctuations of estradiol and progesterone levels during perimenopause. However, our findings do not suggest a continuous decline during the observed transition phase, implying relatively stable periods of fluctuating hormone levels. Furthermore, given the lack of significant group differences, it may not be necessary to differentiate between early and late perimenopause from the standpoint of hormonal progression.

## Introduction

Perimenopause represents the final reproductive transition phase in a woman's life, shifting from the reproductive to non-reproductive state (1). On an average, this period lasts for 2–5 years up to the final menstrual period (FMP), with substantial individual variance in duration (2). The early menopausal stage is characterized by an increased variability in the length of the menstrual cycle, whereas in the late menopausal stage, transitory amenorrhea for 60 days or more (oligomenorrhoea) is prominent (3). During perimenopause, profound hormonal changes occur due to the decline in ovulatory function, resulting in irregular cycles and ultimately the complete cessation of menses, which marks the onset of menopause (2). These endocrine changes are particularly associated with a decrease in gonadal steroid hormones such as estradiol (E2) and progesterone (4). In contrast to the declining levels of E2 and progesterone with respect to the FMP, there is an increase in the steroid hormone cortisol, an outcome marker of the hypothalamic–pituitary–adrenal (HPA) axis (5). The hypothalamic–pituitary–gonadal (HPG) axis and the HPA axis are intertwined such that changes in E2 and progesterone can influence the HPA axis and ultimately, cortisol (5, 6).

Since perimenopause is a protracted process of changing and interrelated hormones, longitudinal approaches are needed to provide further and more detailed insight. The necessity for such studies has long been recognized. Indeed, previous studies have already reported longitudinal findings on the prominent endocrine parameters (4, 7) measured annually and/or two times a year for up to 12 years. Moreover, there are even findings on daily pregnanediol glucuronide (PDG) levels averaged over 6-month intervals for 5 consecutive years (8). The merit of these first studies lies in the knowledge gained about temporary changes in E2, progesterone, and PDG, with all of the studies concluding that the hormone levels decrease with the advancement of perimenopause, particularly in the late menopausal stage. In light of the analyses of more closely spaced, longitudinal data, the assumption of a gradual decline in E2 and progesterone leading up to the FMP has to be reconsidered since the first indications of endocrine fluctuations during the menopausal transition were reported (6, 9, 10). For instance, a study measuring the hormonal levels four times within 14 months indicated a variability of E2 in peri- and post-menopausal women (9). Furthermore, a study measuring the reproductive hormones estrone (E1) and PDG on a daily basis, even reported day-to-day variability in these hormones (10). The study by Gordon et al. (6) revealed not only fluctuations but even increases, in E2 levels around the FMP, when measured weekly, further contributing to the complicated and contradictory picture of endocrine changes within perimenopause.

Although these previous longitudinal studies provided valuable insight into the changes in endocrine parameters throughout the menopausal transition, they also encompass several limitations which impede a further in-depth understanding of the perimenopause and potentially contribute to the contradictory results. First, the study samples often included pre-menopausal women, as well as peri- and/or post-menopausal women (4, 7–9), making it challenging to distinguish valuable information and findings regarding hormonal markers between reproductive and non-reproductive phases. Second, although female transition over several years was investigated in these studies (4, 7), steroid hormones were often measured only one time or two times a year. Such time gaps between measurements complicate the detection of potential fluctuations in these parameters during the menopausal transition, as indicated by long-term studies with repeated measurements taken in close succession (6, 9, 10).

The Swiss Perimenopause Study addresses these limitations by employing closely spaced, longitudinal, and continuous measurements within highly standardized assessments of endocrine changes, measuring E2, progesterone, and cortisol several times for three non-consecutive months over the course of 1 year in solely perimenopausal women. Based on a recent cross-sectional study conducted by our work group (11), which reported fluctuations in E2 and progesterone levels even within one month, the aim of the present study is to contribute to a better understanding of the specific perimenopausal secretion patterns of steroid hormones by illustrating the fluctuations of E2, progesterone, and cortisol longitudinally and continuously over the course of 1 year in 127 perimenopausal women.

## Materials and Methods

The present study was conducted within the scope of the Swiss Perimenopause Study, a large, single-center, and longitudinal research project conducted by our work group as part of the University Research Priority Program Dynamics of Healthy Aging at the University of Zurich, Switzerland. The study was approved by the Cantonal Ethics Committee (KEK-ZH-Nr. 2018-00555) and conducted in accordance with the principles of the Declaration of Helsinki. One central aim of the project was to measure and analyze a variety of biopsychosocial factors associated with menopausal transition over time. The present study is a longitudinal investigation of steroid hormone secretion patterns throughout all biological assessment points, namely during months 2, 7, and 12 [for a detailed description of the study protocol, see (12)].

### Participants and Procedure

The study sample consisted of 127 perimenopausal women aged 40–56 years, who participated in the Swiss Perimenopause Study. All participants were recruited between June 2018 and December 2019, under standardized inclusion and exclusion criteria and provided informed consent prior to assessment. Inclusion criteria to be met at the time of study enrolment were perimenopausal status, as determined by the Stages of Reproductive Aging Workshop (STRAW) criteria (3), and age between 40 and 60 years. Additionally, all perimenopausal women had to self-report good to excellent physical and mental health, which was further confirmed using the German version of the Structured Clinical Interview for the Diagnostic and Statistical Manual of Mental Disorders, fourth edition (DSM-IV) [SCID; (13)] at the time of study enrolment.

Participants reporting psychiatric or psychotropic drug use, consumption of >2 standard units of alcohol per day, hysterectomy or oophorectomy, use of oral contraceptives, pregnancy, or hormone therapy in the last six months were further excluded. Each participant was invited to our lab, where a strictly standardized protocol took place starting at 07:45 am. Among other biophysiological assessments such as bioelectrical impedance analysis and blood sampling during the lab visit, the participants received instructions on saliva sampling verbally, in writing, and on video, as they were asked to independently collect the saliva samples at home over the course of study participation. From the original sample of 135 participants of the Swiss Perimenopause Study, six women withdrew from the project due to the involved effort and were therefore excluded from the analyses. One participant was excluded due to class II obesity (BMI > 35 kg/m^2^) and another participant due to the use of progesterone gel.

### Assessment of Biomarkers

A total of 1,778 saliva samples, 14 samples per participant, were collected every 4th day under standardized conditions for two non-consecutive months (during months 2 and 12) to assess E2 (pg/ml) and progesterone (pg/ml). The participants gathered saliva samples upon awakening. The cortisol awakening response (CAR; ng/ml) and fluctuations of cortisol (μg/dL) throughout the day were measured with a total of 1,524 saliva samples (on awakening, +30 min, +60 min, at 12:00 p.m., and before going to bed), consisting of 10 samples per participant for months 7 and 12 and one additional sample for month 2 (on awakening). These measurement time points were chosen to ensure longitudinal yet closely spaced analyses of endocrine courses during a 12-month period within perimenopause. Month 2 represents the initial start of sample collection for participants, while month 12 represents the end of study participation. For the assessment of endocrine parameters in the middle of study participation, month 7 was chosen for endocrine analyses [for a detailed description of the study protocol, see (12)]. All measurement time points were standardized for all participants regardless of their menopausal stage.

The participants gathered saliva samples using the passive drool method in SaliCap sampling tubes with 2 mL capacity (IBL International GmbH, Hamburg, Germany). Saliva sampling is considered an attractive alternative to blood sampling because of its practicality and noninvasiveness (14). Moreover, the salivary assessment of steroid hormones can be considered as a valid and adequate surrogate marker for serum steroid levels (14, 15). The participants were instructed to store the samples in their own freezers until returning them during their second laboratory assessment. All the saliva samples were subsequently stored at −20°C at the laboratory of the University of Zurich. After thawing, the saliva samples were centrifuged and biochemically analyzed. Hormonal analyses were conducted using enzyme-linked immunosorbent assay (ELISA; IBL International GmbH, Hamburg, Germany). Sensitivity was 0.30 pg/mL for E2, 2.24 pg/mL for progesterone, and 0.003 μg/dL for cortisol. Individual values below the sensitivity threshold were excluded from the analyses (E2, *n* = 66; progesterone, *n* = 17; cortisol, *n* = 3).

### Data Analysis

All analyses were conducted using R statistical software (version 1.3.1.093; R Foundation of Statistical Computing, Vienna, Austria) with the additional packages, “nlme” (16) and “ggplot2” (17). Multilevel analyses were conducted to analyze hormonal changes over time during perimenopause. Multilevel analyses offered various advantages over other types of analysis, such as repeated measures analysis of variance (ANOVA) for longitudinal data, introducing pairwise instead of listwise deletion or imputation of missing data, modeling individual change and variance, or non-linear change in individuals (18).

First, all variables were checked for outliers using graphical plots to depict courses within persons. One participant had to be excluded from the E2 analyses due to implausibly high hormone levels (E2 ≥ 149 pg/mL). Second, five successive models for each endocrine parameter were analyzed and tested to ensure the best model fit. Starting with the basic model including a fixed intercept (model 1), a random intercept (model 2) was introduced, resulting in model 2 before comparison. If model 2 provided a significantly better fit to the data, wherein hormone courses varied over individuals, then multilevel analysis was conducted in line with the recommendations (19). Therefore, to account for the longitudinal data structure, time as a fixed effect (model 3) was introduced to the analyses, followed by random slopes (model 4) to test for individual slopes of hormone courses, and first-order autoregressive covariance structure (model 5). Missing values were excluded pairwise prior to hormonal analyses (E2, *n* = 27; progesterone, *n* = 15; cortisol, *n* = 25). In addition, the courses of E2, progesterone, and cortisol were plotted and analyzed separately for subjects in early and late menopausal transition.

## Results

### Sample Characteristics

Table 1 shows the descriptive characteristics of the sample. The majority of the women were Swiss (86.6%) or German (12.6%). Overall, the sample reported a high level of education (43.3% reported a University degree). The mean age at the time of study enrolment was 48.47 years, and 46.45% of the participants were in early menopause, while 53.55% were in late menopause. Over the duration of the study, 11 women transitioned from the early to the late stage, resulting in 37.80% early and 62.20% late menopausal women at the end of study participation (month 12).

**Table 1:** Descriptive characteristics of the study sample (*n* = 127).

	***N***	***M (SD)***
**Age**		48.47 (3.95)
Age early MT		46.56 (3.97)
Age late MT		50.13 (3.11)
**Menopausal stage month 2**		
Early MT	59	
Late MT	68	
**Menopausal stage month 12**		
Early MT	48	
Late MT	79	
BMI month 2		23.25 (3.45)
BMI month 12		22.51 (3.59)

*BMI, body mass index; M, mean; MT, menopausal transition; N, sample size; SD, standard deviation*.

### Descriptive Characteristics of the Endocrine Parameters

**Figure 1:**
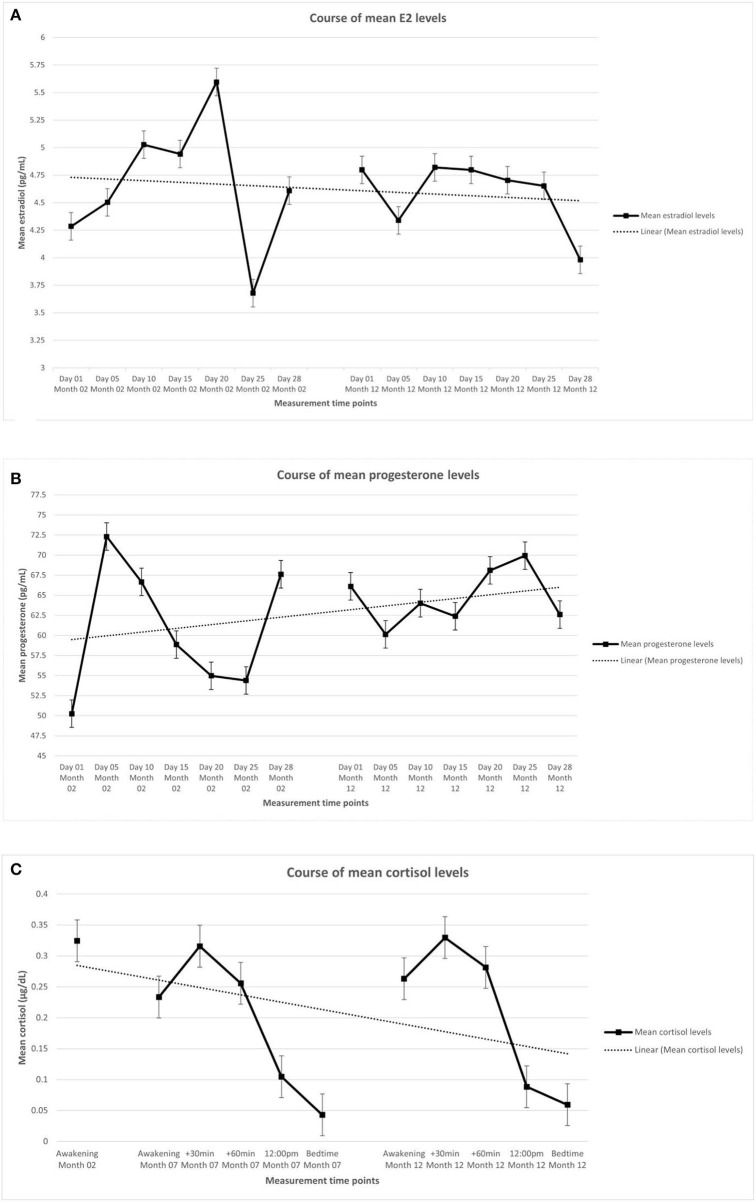
Mean levels of endocrine parameters with linear trend lines and standard error of the mean of participants (*n* = 127). **(A)** Estradiol. **(B)** Progesterone. **(C)** Cortisol. The displayed mean levels of estradiol and progesterone are based on 14 measurements across two non-consecutive months of standardized saliva sampling. Samples 1 to 7 were drawn during month 2 of study participation, samples 8 to 14 were drawn during month 12 of study participation. The displayed mean levels of cortisol are based on 11 measurements across three non-consecutive months of standardized saliva sampling. Sample 1 was drawn during month 2, samples 2 to 6 were drawn during month 7, and samples 7 to 11 were drawn during month 12 of study participation.

**Figure 2:**
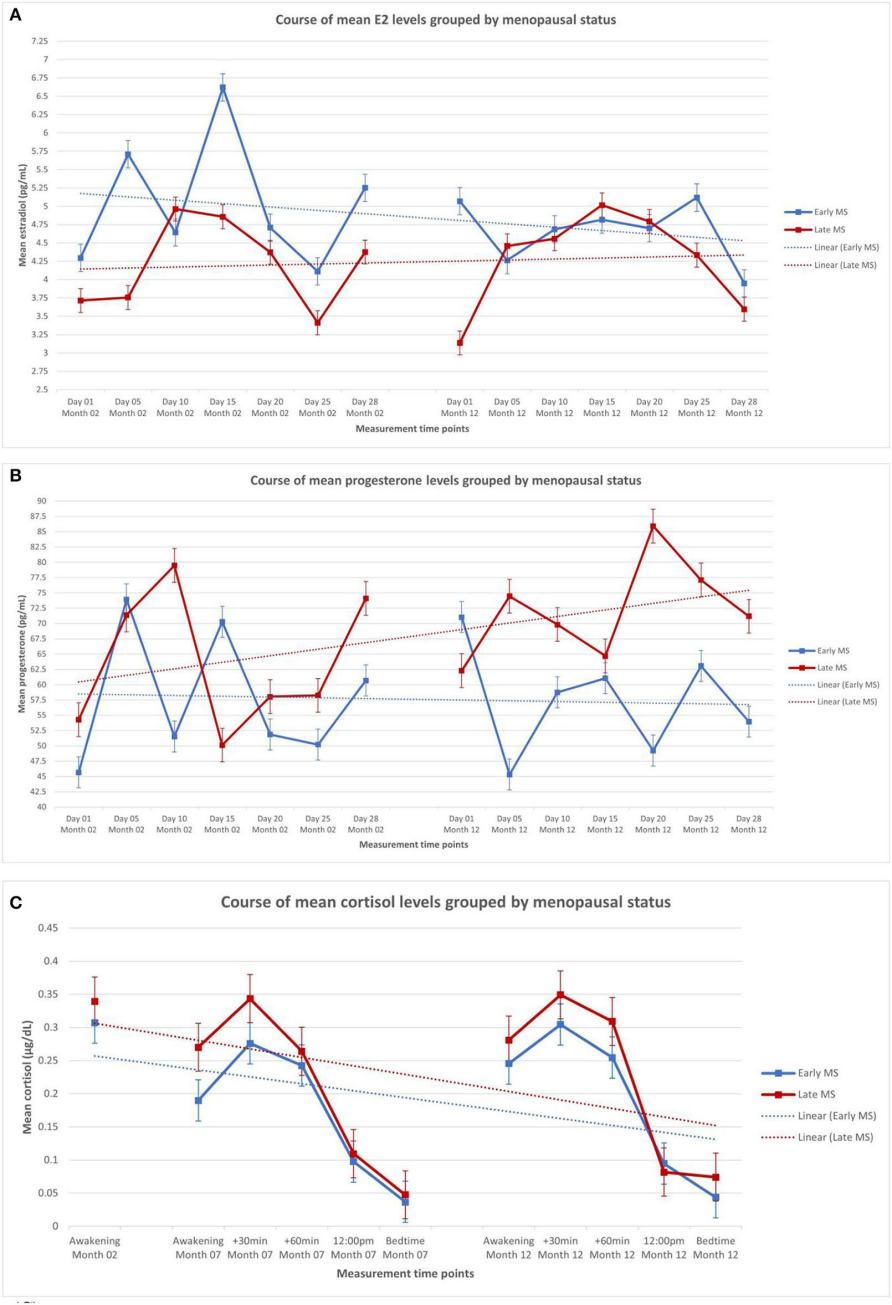
Mean levels of endocrine parameters with linear trend lines and standard error of the mean of participants in the early (*n* = 59) and late perimenopause (*n* = 68). **(A)** Estradiol in early and late perimenopausal participants. **(B)** Progesterone in early and late perimenopausal participants. **(C)** Cortisol in early and late perimenopausal participants. The displayed mean levels of estradiol and progesterone are based on 14 measurements across two non-consecutive months of standardized saliva sampling. Samples 1 to 7 were drawn during month 2 of study participation, and samples 8 to 14 were drawn during month 12 of study participation. The displayed mean levels of cortisol are based on 11 measurements across three non-consecutive months of standardized saliva sampling. Sample 1 was drawn during month 2, samples 2 to 6 were drawn during month 7, and samples 7 to 11 were drawn during month 12 of study participation.

Table 2 shows the descriptive statistics of the endocrine parameters across all 14 measurement time points for E2 and progesterone, and all 11 measurement time points for cortisol, in total and separately for women in early and late menopausal transition. Figure 1 depicts the mean courses of E2, progesterone, and cortisol, and Figure 2 depicts these courses separately for early and late menopausal women. Supplementary Figure 1 further depicts the participants' individual courses of E2, progesterone, and cortisol separately for early and late menopausal women.

**Table 2:** Descriptive statistics of the assessment of estradiol and progesterone across 14 time points over two non-consecutive months and of cortisol across 11 time points over three non-consecutive months, in total and separately, for early and late menopausal women.

	**E2 (pg/mL)**			**Progesterone (pg/mL)**			**Cortisol (μg/dL)**		
	**(***N*** = 126)**			**(***N*** = 127)**			**(***N*** = 127)**		
	**Early MT** **M (SD)**	**Late MT,** **M (SD)**	**Total** **M (SD)**	**Early MT** **M (SD)**	**Late MT** **M (SD)**	**Total** **M (SD)**	**Early MT,** **M (SD)**	**Late MT** **M (SD)**	**Total** **M (SD)**
Total	4.86 (6.17)	4.38 (6.87)	4.63 (6.99)	79.19 (98.29)	55.97 (81.17)	62.52 (88.47)	0.22 (0.28)	0.22 (0.24)	0.21 (0.23)
t1	4.23 (4.30)	4.23 (7.66)	4.28 (6.81)	60.06 (49.31)	48.44 (48.54)	50.25 (47.44)	0.35 (0.37)	0.34 (0.32)	0.32 (0.29)
t2	5.63 (6.78)	3.73 (4.53)	4.50 (5.75)	77.79 (98.51)	70.51 (104.77)	72.31 (106.72)	0.26 (0.31)	0.24 (0.27)	0.23 (0.24)
t3	4.58 (5.24)	5.24 (8.44)	5.03 (7.41)	75.70 (94.62)	66.35 (122.17)	66.67 (111.62)	0.33 (0.29)	0.32 (0.24)	0.32 (0.24)
t4	6.54 (15.62)	3.43 (4.30)	4.94 (11.18)	77.56 (118.23)	48.49 (52.67)	58.87 (87.20)	0.24 (0.17)	0.27 (0.17)	0.26 (0.17)
t5	4.68 (4.48)	5.95 (13.78)	5.60 (11.71)	65.26 (66.74)	49.75 (51.99)	54.98 (58.32)	0.11 (0.10)	0.10 (0.08)	0.11 (0.09)
t6	4.06 (4.12)	3.37 (3.66)	3.68 (3.99)	62.57 (57.16)	51.42 (58.80)	54.40 (55.46)	0.04 (0.03)	0.05 (0.07)	0.04 (0.06)
t7	5.18 (4.84)	4.32 (5.72)	4.61 (5.50)	74.66 (78.45)	63.10 (114.96)	67.62 (107.23)	0.29 (0.39)	0.27 (0.30)	0.26 (0.29)
t8	5.15 (5.84)	4.57 (6.15)	4.80 (6.32)	91.61 (135.42)	51.67 (61.59)	66.12 (97.33)	0.32 (0.23)	0.34 (0.26)	0.33 (0.22)
t9	4.20 (2.43)	4.45 (4.77)	4.34 (4.30)	89.31 (131.98)	56.06 (98.87)	60.13 (93.02)	0.26 (0.19)	0.30 (0.21)	0.28 (0.20)
t10	4.54 (2.95)	4.72 (6.67)	4.82 (6.02)	100.63 (134.53)	49.92 (61.05)	64.01 (94.35)	0.08 (0.08)	0.10 (0.09)	0.09 (0.08)
t11	4.60 (3.58)	4.62 (7.32)	4.80 (6.68)	91.29 (92.03)	51.60 (59.45)	62.39 (74.11)	0.09 (0.32)	0.04 (0.06)	0.06 (0.20)
t12	4.80 (3.91)	4.51 (5.99)	4.70 (5.62)	94.73 (145.76)	54.19 (80.30)	68.11 (110.94)	–	–	–
t13	5.37 (4.61)	4.10 (5.72)	4.65 (5.71)	99.24 (99.44)	60.18 (82.46)	69.93 (85.82)	–	–	–
t14	4.04 (2.61)	3.88 (4.50)	3.98 (4.07)	78.12 (87.57)	62.30 (89.77)	62.60 (82.23)	–	–	–

*E2, estradiol; M, mean; MT, menopausal transition; N, sample size; SD, standard deviation*.

### Multilevel Analyses of Hormonal Courses

#### Course of E2 During Perimenopause

To analyze the course of E2 during perimenopause, nested multilevel models were conducted and compared for best model fit as described in the section Data analysis. The baseline model (model 1) included a fixed intercept predicting E2 levels. Model 2 with a random intercept was then compared with model 1 in terms of model fit. Model 2 provided a significantly better fit to the data [$\chi_{\left(1\right)}^{2}$ = 249.66, *p* < 0.0001], but the inclusion of time as a fixed effect (model 3) did not significantly increase the model fit [$\chi_{\left(1\right)}^{2}$ = 0.18, *p* > 0.05]. Model 4, which analyzed the course by including random slope, random intercept, and time as a fixed effect, significantly enhanced the model fit, $\chi_{\left(2\right)}^{2}$ = 41.66, *p* < 0.0001, thereby resulting in the final model. Introducing first-order autoregressive covariance structure (model 5) to the analysis did not significantly enhance the fit, $\chi_{\left(1\right)}^{2}$ = 1.61, *p* > 0.05. In the final model (model 4), E2 levels showed significant variance in intercepts across participants over time [*SD* = 5.16 (95% CI: 4.28, 6.21)]. Additionally, the slopes varied across participants over time [*SD* = 0.50 (95% CI: 0.39, 0.64), $\chi_{\left(2\right)}^{2}$ = 41.66, *p* < 0.0001]. Intercepts and slopes were negatively and significantly correlated [cor = −0.70 (−0.91, −0.53)]. Time did not significantly predict E2 levels [*b* = −0.01, *t*_(1388)_ = −0.21, *p* > 0.05].

#### Course of Progesterone During Perimenopause

Nested multilevel models were calculated and compared in order to analyze the course of progesterone during perimenopause, as described in the section Data analysis. The baseline model (model 1), including a fixed intercept predicting progesterone levels, was compared with model 2, including a random intercept. Model 2 showed a significantly improved model fit compared to model 1 [$\chi_{\left(1\right)}^{2}$ = 420.61, *p* < 0.0001]. Model 3, adding a fixed time effect, did not provide a significantly better fit than model 2 [$\chi_{\left(1\right)}^{2}$ = 2.53, *p* > 0.05]. Both model 4, adding a random slope [$\chi_{\left(2\right)}^{2}$ = 43.92, *p* < 0.0001], and model 5, adding an autoregressive covariance structure [$\chi_{\left(1\right)}^{2}$ = 4.58, *p* < 0.0001] significantly represented the data better, with model 5 being chosen as the final model. Progesterone levels showed significant variance in intercepts [*SD* = 34.77 (95% CI: 25.55, 47.31)] and slopes [*SD* = 4.17 (95% CI: 2.91, 5.99)] across participants over time. Intercepts and slopes were correlated positively but not significantly [cor = 0.65 (−0.26, 0.95)]. Time did not significantly predict progesterone levels [*b* = −0.01, *t*_(1, 388)_ = −0.21, *p* > 0.05].

#### Course of Cortisol During Perimenopause

The course of cortisol during perimenopause was analyzed using nested multilevel models, as described in the section Data analysis. Model 1, the baseline model, predicted the cortisol levels with a fixed intercept. Model 1 was compared to model 2 with a random intercept for better model fit. The random intercept model (model 2) significantly enhanced the model fit [$\chi_{\left(1\right)}^{2}$ = 13.87, *p* < 0.0001]. Model 3 with a fixed time effect provided a significantly better fit to the data compared to model 2 [$\chi_{\left(1\right)}^{2}$ = 63.28, *p* < 0.0001], as did model 4 with a random slope [$\chi_{\left(2\right)}^{2}$ = 46.81, *p* < 0.0001]. The final model 5, introducing an autoregressive covariance structure, further improved the fit significantly [$\chi_{\left(1\right)}^{2}$ = 80.30, *p* < 0.0001]. Significant variance in intercepts [*SD* = 0.18 (95% CI: 0.14, 0.23)] and slopes [*SD* = 0.02 (95% CI: 0.01, 0.02)] across participants over time were found for cortisol levels. The two random effects, intercept and slope, were negatively and significantly correlated [cor = −0.99 (−1.00, −0.75)]. Time significantly predicted cortisol levels [*b* = −0.02, *t*_(979)_ = −6.63, *p* < 0.0001].

### Endocrine Courses in Early and Late Perimenopause

Nested multilevel models, as described in the section Data analysis, were used to analyze the influence of perimenopausal state on E2, progesterone, and cortisol courses. The perimenopausal stage (early vs. late) was therefore added as a predictor variable for E2, progesterone, and cortisol courses. Model 5, with a random intercept [E2: *SD* = 5.27 (95% CI: 4.42, 6.29), $\chi_{\left(1\right)}^{2}$ = 301.48, *p* < 0.0001; progesterone: *SD* = 40.32 (95% CI: 32.06, 50.71), $\chi_{\left(1\right)}^{2}$ = 540.67, *p* < 0.0001)] and random slope [E2: *SD* = 0.53 (95% CI: 0.42, 0.65), $\chi_{\left(2\right)}^{2}$ = 59.78, *p* < 0.0001; progesterone: *SD* = 4.92 (95% CI: 3.78, 6.41), $\chi_{\left(2\right)}^{2}$ = 62.79, *p* < 0.0001)], proved to be the best-fitting model for E2 and progesterone. Perimenopausal state did not significantly predict E2 scores over time [*b* = −0.36, *t*_(1608)_ = −0.84, *p* = 0.400] or progesterone scores over time [*b* = −4.55, *t*_(1723)_ = −0.87, *p* = 0.385]. For cortisol, model 6 with a random intercept [*SD* = 0.22 (95% CI: 0.19, 0.26), $\chi_{\left(1\right)}^{2}$ = 267.19, *p* < 0.0001], random slope [*SD* = 0.02 (95% CI: 0.01, 0.02), $\chi_{\left(2\right)}^{2}$ = 102.84, *p* < 0.0001], and autoregressive covariance structure showed the best fit. Perimenopausal status showed no significant predictive effect on cortisol values over time [*b* = 0.01, *t*_(1, 124)_ = 0.61, *p* = 0.542].

## Discussion

The current study investigated the secretion patterns of steroid hormones in perimenopausal women in a highly standardized manner. The results showed that for E2 and progesterone, courses over time significantly differed interindividually, and fluctuations were evident at every measurement time point. Nonetheless, neither time nor early vs. late perimenopause predicted E2 and/or progesterone levels significantly, indicating that no general decrease or increase over time was apparent. Interindividual differences were also discernible for the course of cortisol. Time of day showed a significant negative effect on the cortisol levels, whereas perimenopausal stage was unrelated to the cortisol levels.

Recent findings suggest highly individual fluctuations in E2 and progesterone during perimenopause (6, 9, 11). As such, the current results are consistent with previous findings of fluctuating secretion patterns of sex steroids during perimenopause, and contradict findings of a continuous decline due to the perimenopausal transition (4, 7). The short intervals between measurements applied in the present study could have enabled the detection of short-term fluctuations. Our results could further indicate that within the aforementioned continuous decline during perimenopause, somewhat stable periods of fluctuating hormone levels are observable, during which women in early vs. late perimenopause do not show differences in their E2 and progesterone courses. Accordingly, late perimenopause does not seem to represent exclusive characteristics of E2 and progesterone courses compared to early perimenopause, which is contrary to previous reports that the amplitude of fluctuations of E2 and progesterone levels increases during late perimenopause (20). This discrepancy between our results and the finding of a significant difference in fluctuation amplitude within sex steroids between early and late perimenopause could be attributed to the non-standardized usage of classification manuals for perimenopausal stages within different longitudinal studies. Additionally, only a small proportion of our sample transitioned from early to late perimenopause during the time of study participation, whereas all the other women remained within their initial stage. Future longitudinal studies, which include early perimenopausal women at study enrolment and assess them throughout their individual transition to late perimenopause, could foster our understanding of the impact of intra- and interindividual factors on different endocrine courses.

Cortisol levels have been suggested to fluctuate over time, with the daily cortisol secretion increasing with age (21). While our results are consistent with previous reports of interindividual fluctuations of cortisol levels, we did not find increased cortisol levels over the three assessment days. The time-related decrease in cortisol levels can likely be explained by diurnal trends: Over the course of a day, the natural cortisol level decreases (22), and since the cortisol levels were measured up to five times per day during study participation, our results reflect this diurnal trend. Furthermore, subjective stress levels and vasomotor symptoms, which are usually associated with increased cortisol levels, could have been less present in our study sample given that we were investigating a healthy population (23). The lack of association between cortisol courses and perimenopausal stage in the present study is also consistent with previous studies linking cortisol levels to age but not to early vs. late perimenopause (23, 24).

The present findings should be interpreted in the light of some limitations. The ability to generalize our results is limited due to the strict inclusion criteria and the specific study population assessed, which led to a relatively homogeneous study population. Furthermore, participants collected saliva samples depending on their respective time of awakening. Consequently, a variability in the time of sampling is evident, thereby potentially affecting endocrinological parameters through irregular sleep/wake cycles and/or sleep disturbances. The fact that there is currently no consensus regarding quantitative E2 and progesterone levels further complicates the interpretation and comparability of our results. The high heterogeneity of reported sex steroid levels is already evident by the fact that for E2 levels in peri- and post-menopausal women alone, mean values ranging from 55.08 pg/mL (4) to 127.5 pg/mL (9) can be found in serum analyses and 1.31 pg/mL (6) to 1.89 pg/mL (25) in saliva analyses. Indeed, the need for reference ranges for sex steroids that are derived from studies with adequate sample sizes and standardized procedures is one of the greatest challenges (26), to our subsequent understanding of the phases characterized by biological upheaval. Although the salivary assessment of steroid hormones is seen as a valid surrogate for blood sampling with correlations ranging from 0.74 to 0.80 for E2 levels (14, 27), salivary assessments have not been shown to be a reliable medium when measuring very low E2 levels (28, 29) as can be found in post-menopausal women (30) and men (29). The assessment method used therefore further complicates the comparability of our results with other studies using different assessment methods. Nevertheless, saliva collection offers several advantages over blood collection, such as the non-invasive approach, practicality, and ease of using saliva samples in a longitudinal design with repeated measures (14).

Besides these limitations, this study also has several strengths, the most salient of which is probably the inclusion of solely perimenopausal women with a standardized classification of menopausal stage through the usage of the STRAW criteria. Moreover, due to our strict inclusion criteria, the study population consisted of physically and mentally healthy women at the time of study enrolment. The assessments of endocrine markers were conducted in close succession and spread over the period of 1 year of the perimenopausal transition, allowing for a more detailed depiction of the hormonal courses.

## Conclusion

Our study results contradict the assumption of a continuous decline in E2 and progesterone during the menopausal transition assessed over a 12-month period within perimenopause. This may be indicative of somewhat stable periods of fluctuating hormone levels within a period of a woman's life, which is normally associated with a continuous decrease in sex steroid levels. Furthermore, this continuous decline was neither observable among the overall study population nor among the early or late perimenopausal women separately. Consequently, separate endocrine analyses for early and late perimenopause may not be necessary from the standpoint of hormonal progression due to a lack of significant group differences.

## Data Availability Statement

The raw data supporting the conclusions of this article will be made available by the authors, without undue reservation.

## Ethics Statement

The studies involving human participants were reviewed and approved by Cantonal Ethics Committee (KEK-ZH-Nr. 2018-00555). The patients/participants provided their written informed consent to participate in this study.

## Author Contributions

JG developed the concept of this manuscript and was responsible for the data acquisition and drafting of the manuscript. HS and JW were responsible for data acquisition as well as critically reviewing the manuscript. UE developed the concept and provided critical revision of the manuscript. All the authors have read and approved the final manuscript.

## Funding

This research was funded by the University of Zurich Research Priority Program (URPP) Dynamics of Healthy Aging. The funding body was not involved in the design of the study, the collection, analysis, and interpretation of the data, or in the writing of the manuscript.

## Conflict of Interest

The authors declare that the research was conducted in the absence of any commercial or financial relationships that could be construed as a potential conflict of interest.

## Publisher's Note

All claims expressed in this article are solely those of the authors and do not necessarily represent those of their affiliated organizations, or those of the publisher, the editors and the reviewers. Any product that may be evaluated in this article, or claim that may be made by its manufacturer, is not guaranteed or endorsed by the publisher.
