# Supplementary material for: Steroid Hormone Secretion Over the Course of the Perimenopause: Findings From the Swiss Perimenopause Study
Source: Front Glob Womens Health. 2021 Dec 14;2:774308. doi: 10.3389/fgwh.2021.774308 (PMC8712488; doi:10.3389/fgwh.2021.774308)
Supplement: Supplementary file 1 [file Data_Sheet_1.PDF]

Supplementary Material

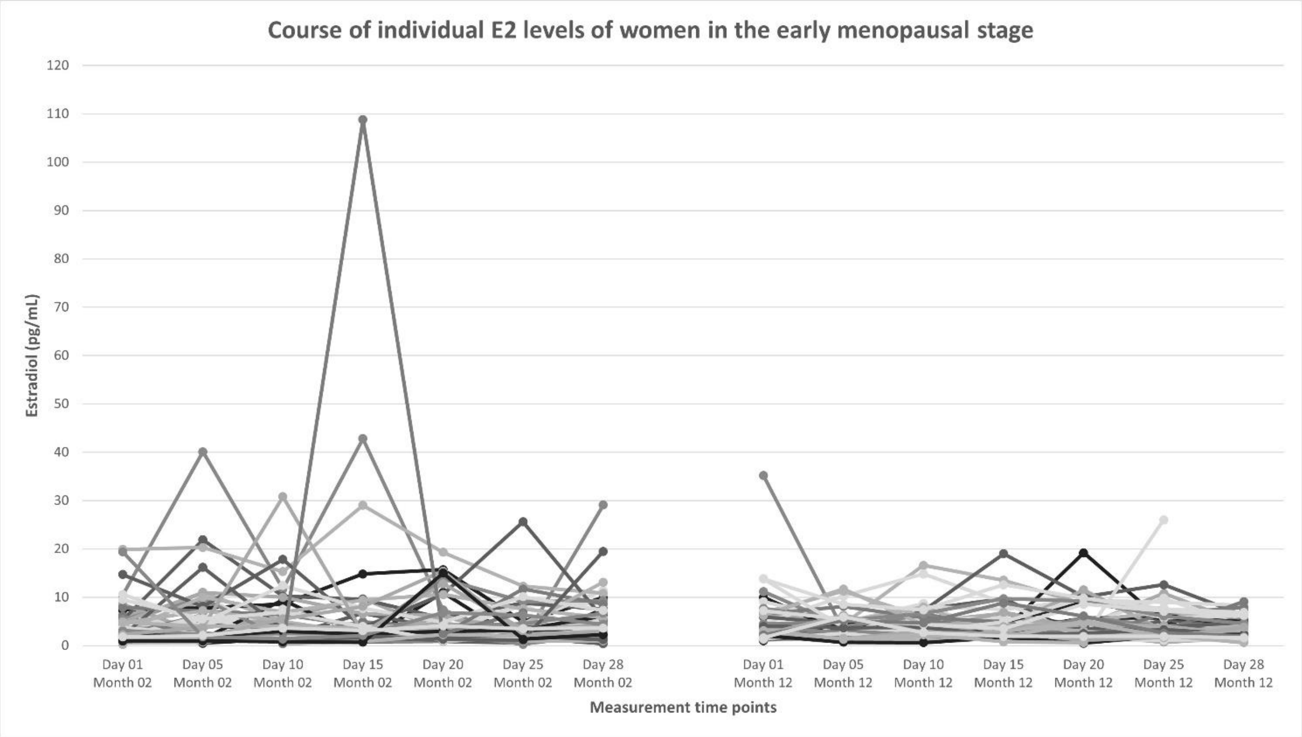

(A)

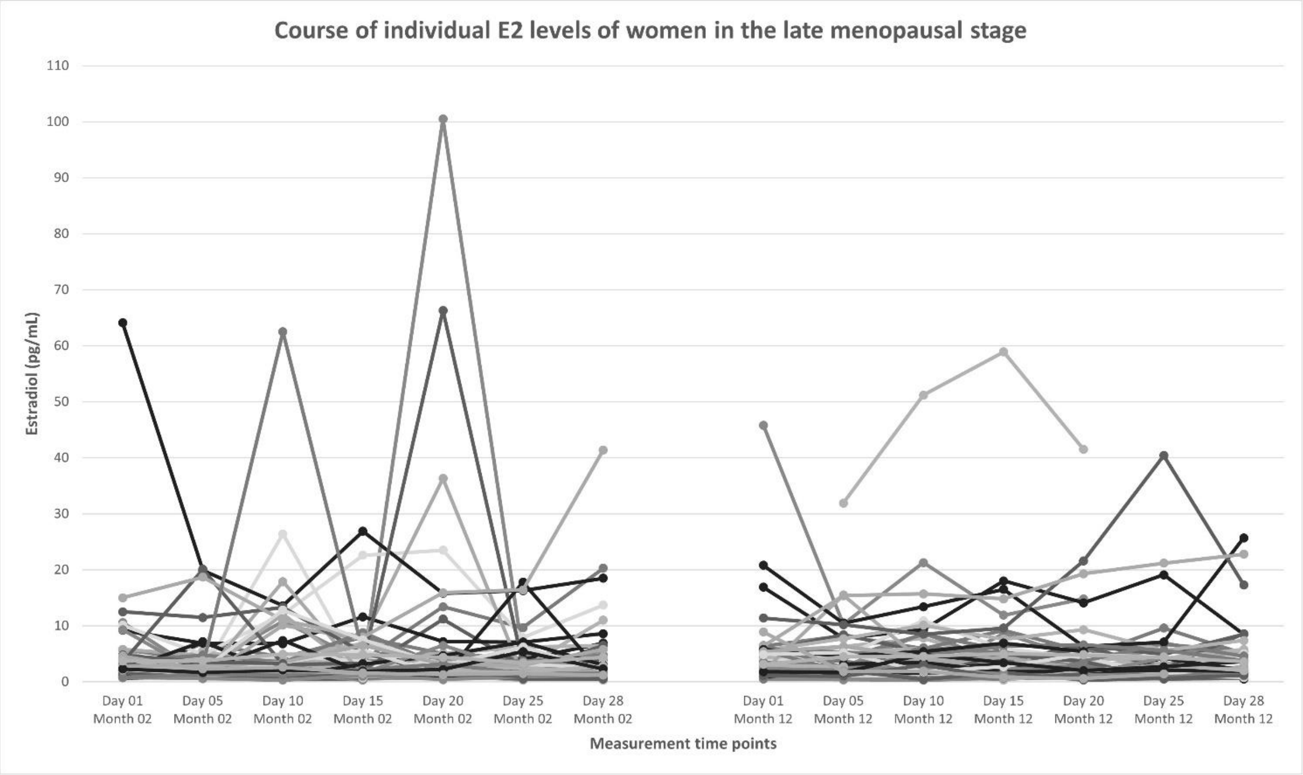
Course of individual E2 levels of women in the late menopausal stage
Estradiol (pg/mL)
110
100
90
80
70
60
50
40
30
20
10
0
Day 01 Month 02
Day 05 Month 02
Day 10 Month 02
Day 15 Month 02
Day 20 Month 02
Day 25 Month 02
Day 28 Month 02
Day 01 Month 12
Day 05 Month 12
Day 10 Month 12
Day 15 Month 12
Day 20 Month 12
Day 25 Month 12
Day 28 Month 12
Measurement time points

(B)

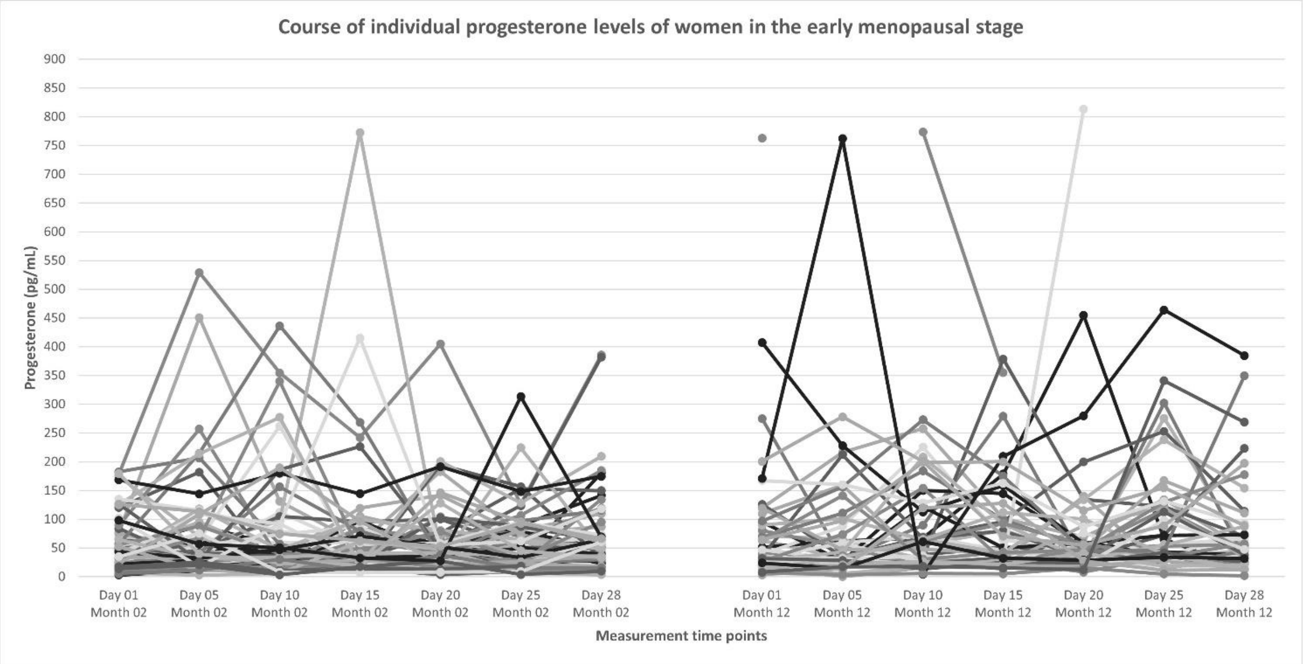
Course of individual progesterone levels of women in the early menopausal stage
Progesterone (pg/mL)
900
850
800
750
700
650
600
550
500
450
400
350
300
250
200
150
100
50
0
Day 01 Month 02
Day 05 Month 02
Day 10 Month 02
Day 15 Month 02
Day 20 Month 02
Day 25 Month 02
Day 28 Month 02
Day 01 Month 12
Day 05 Month 12
Day 10 Month 12
Day 15 Month 12
Day 20 Month 12
Day 25 Month 12
Day 28 Month 12
Measurement time points

(C)

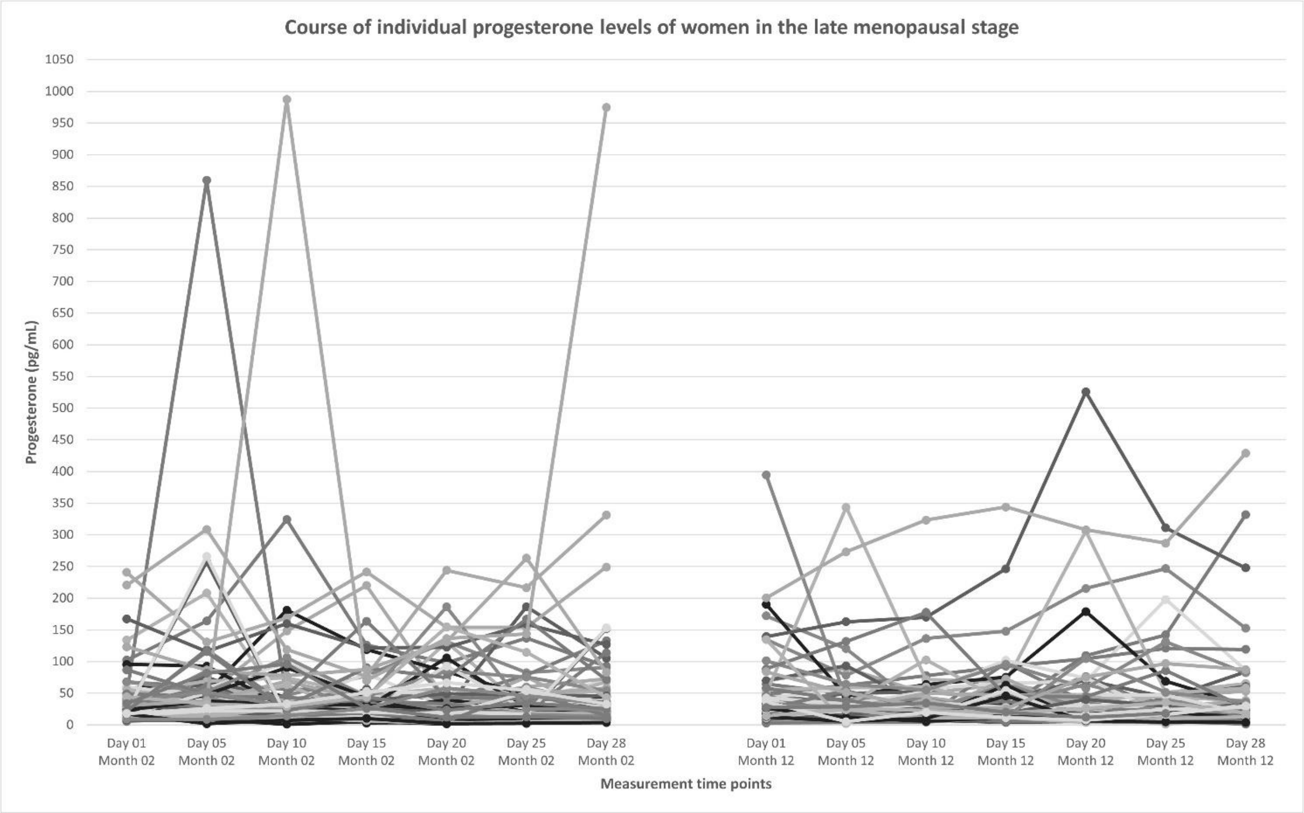
Course of individual progesterone levels of women in the late menopausal stage
Progesterone (pg/mL)
1050
1000
950
900
850
800
750
700
650
600
550
500
450
400
350
300
250
200
150
100
50
0
Day 01 Month 02
Day 05 Month 02
Day 10 Month 02
Day 15 Month 02
Day 20 Month 02
Day 25 Month 02
Day 28 Month 02
Day 01 Month 12
Day 05 Month 12
Day 10 Month 12
Day 15 Month 12
Day 20 Month 12
Day 25 Month 12
Day 28 Month 12
Measurement time points

(D)

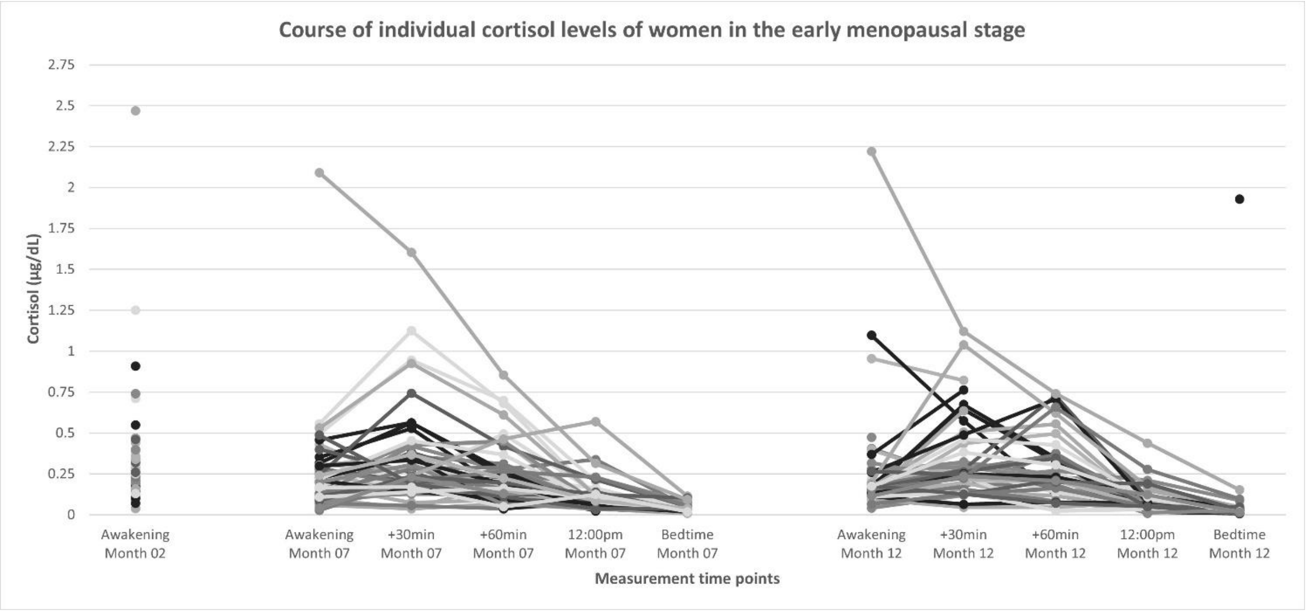
Course of individual cortisol levels of women in the early menopausal stage
Cortisol (µg/dL)
2.75
2.5
2.25
2
1.75
1.5
1.25
1
0.75
0.5
0.25
0
Awakening Month 02
Awakening Month 07
+30min Month 07
+60min Month 07
12:00pm Month 07
Bedtime Month 07
Awakening Month 12
+30min Month 12
+60min Month 12
12:00pm Month 12
Bedtime Month 12
Measurement time points

(E)

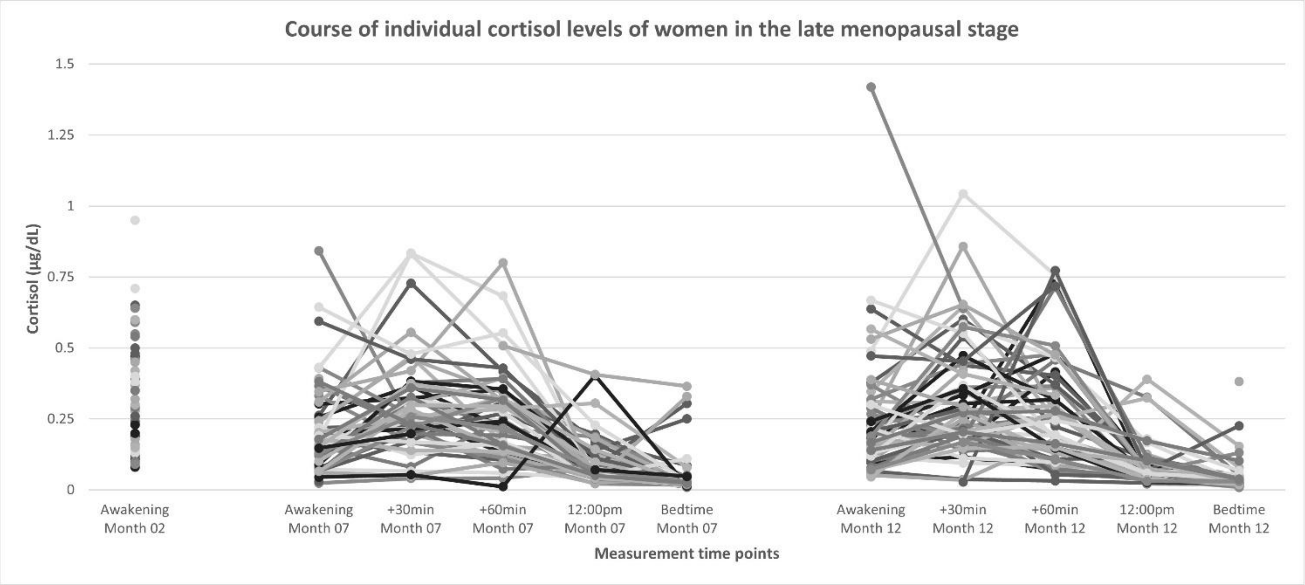

(F)

Supplementary Figure 1. Individual levels of endocrine parameters of participants in the early (n = 59) and late perimenopause (n = 68). **(A)** Estradiol in early perimenopausal participants. **(B)** Estradiol in late perimenopausal participants. (C) Progesterone in early perimenopausal participants. (D) Progesterone in late perimenopausal participants. (E) Cortisol in early perimenopausal participants. (F) Cortisol in late perimenopausal participants. The displayed individual levels of estradiol and progesterone are based on fourteen measurements across two non-consecutive months of standardized saliva sampling. Samples 1 to 7 were drawn during month 2 of study participation, samples 8 to 14 were drawn during month 12 of study participation. The displayed individual levels of cortisol are based on eleven measurements across three non-consecutive months of standardized saliva sampling. Sample 1 was drawn during month 2, samples 2 to 6 were drawn during month 7, and samples 7 to 11 were drawn during month 12 of study participation.
